# Morphological and molecular evidence reveals a new species of chewing louse *Pancola ailurus* n. sp. (Phthiraptera: Trichodectidae) from the endangered Chinese red panda *Ailurus styani*

**DOI:** 10.1016/j.ijppaw.2022.12.004

**Published:** 2022-12-27

**Authors:** Yuan-Ping Deng, Wei Wang, Yi-Tian Fu, Yu Nie, Yue Xie, Guo-Hua Liu

**Affiliations:** a Research Center for Parasites & Vectors, College of Veterinary Medicine, Hunan Agricultural University, Changsha, Hunan Province, 410128, China; b The Centre for Bioinnovation, School of Science and Engineering, University of the Sunshine Coast, Sippy Downs, QLD, 4556, Australia; c Department of Parasitology, College of Veterinary Medicine, Sichuan Agricultural University, Chengdu, Sichuan Province, 611130, China

**Keywords:** Chinese red panda, Chewing lice, *Pancola ailurus*, New species, Systematics

## Abstract

Lice are six-legged, wingless, insect parasites of mammals and birds, and include two main functional groups: blood-sucking lice and chewing lice. However, it is still not clear whether the Chinese red panda *Ailurus styani* is infested with the parasitic louse. In the present study, we describe a new genus and a species of chewing louse, *Pancola ailurus* (Phthiraptera: Trichodectidae) based on morphological and molecular datasets. The morphological features showed that *Pancola* is closer to *Paratrichodectes*. The genetic divergence of *cox*1 and 12S rRNA among the *Pancola ailurus* n. sp. and other Trichodectidae lice was 29.7 – 34.6% and 38.9 – 43.6%, respectively. Phylogenetic analyses based on the available mitochondrial gene sequences showed that *P*. *ailurus* n. sp. is more closely related to *Trichodectes canis* and *Geomydoecus aurei* than to *Felicola subrostratus* and together nested within the family Trichodectidae. This study is the first record of parasitic lice from the endangered Chinese red panda *A*. *styani* and highlights the importance of integrating morphological and molecular datasets for the identification and discrimination of new louse species.

## Highlights

• First record of parasitic lice from *A. styani*.
• *Pancola* is a new genus and *P. ailurus* is a new species.
• First descriptions of this genus and species.

## Graphical abstract

**Figure:**
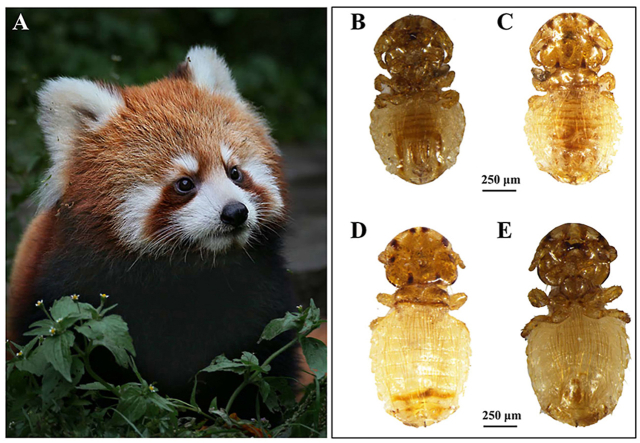

## 1 Introduction

Parasitic lice (Psocodea: Phthiraptera) are permanent, obligate, and host-specific ectoparasites commonly found on the surface of birds and mammals (Kim, 1988; Price et al., 2003). The entire life of the lice is completed on their hosts (including egg, three nymphal, and adult stages). To date, five taxonomic groups of lice are recognized in the world: Anoplura, Amblycera, Ischnocera, Rhynchophthirina, and Trichodectoidea (De Moya et al., 2021). The lice in the groups of Amblycera, Ischnocera, Rhynchophthirina, and Trichodectidea are all chewing lice, which mainly feed on the feathers and dermal debris of their hosts. The Anoplura lice are all sucking lice, which only feed on the host blood (Light et al., 2010). Although chewing lice are relatively benign parasites, the infestation of lice can cause public health impacts. Heavy infestations can cause their hosts’ severe skin irritation, scratching and rubbing, hair loss, fur damage, fleece and wool damage, restlessness, weight loss, and severe anemia (Mullen and Durden 2009; Clayton et al., 2015; Bush and Clayton, 2018). Approximately 400 species of chewing lice in 19 genera are recognized in the family Trichodectidae. The members of Trichodectidae only parasitize eutherian mammals and can be morphologically distinguished by only one tarsal claw on each leg, and the antennae in at least one sex have either a reduced number of segments or the reduced size of the last two segments (Ewing, 1936). Trichodectidae poses severe threats to livestock husbandry worldwide (Price et al., 2003). For instance, the dog-biting louse (*Trichodectes canis*) and the cat-biting louse (*Felicola subrostratus*) are intermediary hosts of the dog tapeworm *Dipylidium caninum*, which not only infect canids but also humans (Dantas-Torres, 2008; Rousseau et al., 2022).

Red pandas (Carnivora: Ailuridae), which are strictly endemic to the Himalaya-Hengduan Mountains mainly live in high altitudes (2200 to 4800 m) (Roberts and Gittleman, 1984; Glatston et al., 2015), are endangered mammals to the world. Based on morphology, biogeography, and population genetic evidence, red pandas are classified into two distinct species within the geographic boundary of the Yalu Zangbu River: *Ailurus fulgens* (Himalayan) and *Ailurus styani* (Chinese) (Hu et al., 2020). Populations of Chinese red pandas have been on the decline by 50% over the past 30 years (Glatston et al., 2015; Karki et al., 2021). The main factors linked to this decline have been hunting and fragmentation, degradation, and destruction of habitats (Pradhan et al., 2001; Thapa et al., 2018; Karki et al., 2021). Another likely threat to the red panda is parasite infections (Sharma and Achhami, 2021). Chinese red pandas have been reported with several parasites including protozoans, cestodes, nematodes, and trematodes (Bertelsen et al., 2010; Bista et al., 2017; Sharma and Achhami, 2021). Interestingly, ticks and mites were also reported (Bista et al., 2017), but no louse species has previously been recorded from the red pandas.

The traditional methods for the identification and differentiation of louse species are based on the morphological features and the relevant information of hosts or geographical origin (Madrid et al., 2020), and the morphological method helped identify and distinguish over 5000 louse species based on the last worldwide checklist (Price et al., 2003). To date, morphology is still considered the mainstream for identifying new species in a lot of studies (Lei et al., 2020; Wang et al., 2020, 2021; Sychra and Palma, 2021; Jie et al., 2022). As a supplementary method, the molecular method to identify and distinguish louse species seems to be helpful for non-experts, and it could expand the understanding of the possible divergence of new species. With the assistants of molecular methods, it is easier to distinguish body lice and head lice that are similar in shape (Fu et al., 2022). Additionally, the molecular method was also developed as a rapid detection of lice infestations (Tran et al., 2022). Moreover, a large literature on both sucking and chewing lice added molecular information as supplementary data when describing new species (Valim and Weckstein, 2013; Najer et al., 2014; Kolencik et al., 2018; Durden et al., 2019; Madrid et al., 2020). Herein, we aim to combine morphological characters with molecular data for better identifying louse species.

## 2 Materials and methods

### 2.1 Specimens collection

**Fig. 1:**
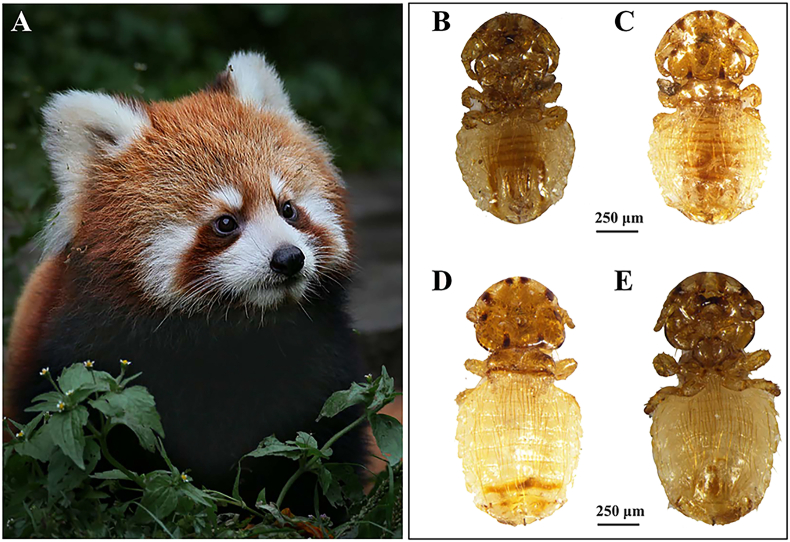
Chinese red panda *Ailurus styani* and the lice collected from its surface. **(A)** the Chinese red panda from Chengdu Research Base of Giant Panda Breeding, Chengdu County, Sichuan Province, China (N30.743°, E104.150°) **(B)** the abdomen of male *Pancola ailurus***(C)** the back of male *P*. *ailurus***(D)** the back of female *P*. *ailurus***(E)** the abdomen of female *P*. *ailurus.*

Louse specimens were collected from Chinese red pandas *A*. *styani* in the Sichuan province of China in November 2020. The collection procedures followed animal ethics and have no damage to both hosts or parasites. Using dandruff combs, the chewing lice on the surface of red pandas were combed down and collected (Fig. 1). The collected lice were washed with physiological saline solution. Some of them were used for morphological examination, specimen collection, and DNA extractions, and the rest were stored at - 80 °C for further use.

### 2.2 Morphological examination

For further morphological examination, clearing, staining, and mounting of intact specimens with minimum content and stretched legs on the slides as Palma detailed (Palma, 1978). With paratergal plates pre-pierced, lice were dipped into 20% potassium hydroxide solution (KOH) for 24 – 48 h until soft, and transparent. After gently squeezing the abdomens with tweezers to expel the digested tissues, lice were transferred into ultrapure water for 30 min. Then lice were transferred into a 10% acetic acid solution for 30 min. After that, the specimens were subsequently stained in 1% acid fuchsin for 4 h, then gradually dehydrated with 40%, 80%, and absolute ethyl alcohol for 30 min, respectively. After finishing the steps above, specimens were immersed in clove oil to purify dying for 1 – 2 weeks. The specimens were then applied on slides with a drop of Canada balsam at room temperature (25 °C) drying for 2 – 3 weeks. The slides were examined under a digital photomicroscope (OLYMPUS BX51). A key to Trichodectidae proposed by Lyal (1985) has been used for identification and comparison. All measurements were taken in micrometers (range followed by mean). Descriptive format and abbreviations of morphological features, with names of setae spelled out in full at first mention, follow Price and Hellenthal (1981), Lyal (1985), Gustafsson and Bush (2017), and Mey (2021).

### 2.3 DNA isolation, amplification, and sequencing

Whole-genome including mitochondrial (mt) and nuclear DNA was extracted from 38 single lice using Wizard® SV Genomic DNA Purification System (Promega, USA) according to the manufacturer introduction. The preliminary morphological identification inferred red panda lice similar to the family Trichodectidae species, and we then designed pairs of primers based on available trichodectids sequences to amplify the nearly complete mt genes (*cox*1 and 12S rRNA) and nuclear gene (18S rRNA) of these lice (Barker et al., 2003; Song et al., 2019), since most of the sequences available online are partial sequences and may not be the same fragments. The primers were listed in Table 1. PCR amplifications used a 2 μl DNA template, 1.5 μl of each primer (forward and reverse), 25 μl of Master Mix (Takara, Shiga, Japan), and added ddH_2_O to a 50 μl system. The amplification conditions were followed as denaturation at 94 °C for 1 min, followed by 37 cycles of denaturation at 98 °C for 10 s, then 30 s of annealing at 45 °C for *cox*1 and 47 °C for 12S and 18S, 1 min of extension at 72 °C, and 2 min of the final extension at 72 °C. PCR products were then verified with 1.5% agarose gel electrophoresis. Purified products were sequenced by the Sanger method in BGI Tech Solutions Co., Ltd. (Shenzhen, China).

**Table 1:** The designed primers were used to amplify the sequences of *Pancola ailurus*.

Primer name	Sequence (5′- 3′)	Amplicon size
18SF	TCTTTCAAATGTCTGACTTATC	∼1700 bp
18SR	TAAATCGTTCAATCGGTAGTAG	
Cox1F	TGTAATAGATATGCTAAAACTG	∼1600 bp
Cox1R	TTCTATCTATCCTATCTCTCCC	
12SF	TTAGAGAATAAAAATAGAAATA	∼1000 bp
12SR	ATTTATCTTACACCCCATTCTT	

### 2.4 Phylogenetic analyses and divergence dating

Phylogenetic relationships among species were inferred based on combined mt sequences (*cox*1 and 12S rRNA) within Trichodectidae and Bovicoliidae (Table 2) using the Maximum likelihood (ML) algorithm, with *Liposcelis bostrichophila* (KY656897) as the outgroup. All nucleotide sequences of families Trichodectidae and Bovicoliidae species were then aligned into a single alignment dataset with ClustalX 1.83 (Aiyar, 2000), and excluded ambiguous regions based on Gblocks 0.91b webserver with the selection of “less stringent” (Castresana, 2000; Dereeper et al., 2008). The most suitable model “GTR + I + G” was selected by jModelTest v2.1.5 with Akaike information criterion (AIC) (Darriba et al., 2012). ML analysis was conducted based on concatenated *cox*1 and 12S rRNA gene sequences in PhyML v3.1 with a nucleotide substitution model. To verify the reliabilities of each inferred phylogeny, 100 bootstraps were applied.

**Table 2:** The chewing lice from Trichodectidae and Bovicoliidae were used in the phylogenetic analysis in this study.

Species	Host	GenBank ID	Reference
*Bovicola ovis*	Sheep	MH001203/MH001211	Song et al. (2019)
*Bovicola caprae*	Goat	MH001178/MH001186	Song et al. (2019)
*Bovicola bovis*	Cattle	MH001191/MH001199	Song et al. (2019)
*Trichodectes canis*	Canine	MH001214/MH001222	Song et al. (2019)
*Trichodectes canis*	Canine		Our own data
*Geomydoecus aurei*	Gopher	KX228450/MW396892	Pietan et al. (2016)
*Damalinia meyeri sika*	Sika deer	JN122004/JN122002	Cameron et al. (2011)
*Felicola subrostratus*	Cat		Our own data
*Felicola subrostratus*	Cat		Our own data

The divergence time of chewing lice from families Trichodectidae and Bovicoliidae was determined using BEAST v1.10.4 (Suchard et al., 2018). Considering the divergence time of the recent common ancestor (*Pediculus humanus* and *P. schaeffi*) used in the present study was inferred mainly based on the *cox*1 gene (Light et al., 2010), phrased partial mt gene (*cox*1) implemented in BEAST v1.10.4 to estimate the BEAST coalescent species. The most suitable model was determined based on ModelFinder with “mtREV” used as the gamma site model. Divergence time analysis was set as followed: a random starting tree, a strict clock model, and a birth-death tree prior with an uncorrelated lognormal clock rate (Drummond et al., 2006). Considering the uncertainty of default calibrations, we introduced members whose divergence dates had been published before: (i) the calibration of booklouse (*L*. *bostrychophila*), which is seen as the closest free-living relative of parasitic lice (Yoshizawa and Johnson, 2010), was considered a potential ancient divergence time in 100 million years ago (Mya) (Grimaldi and Engel, 2006); (ii) a calibration for human lice (*P*. *humanus*) and chimpanzee lice (*P*. *schaeffi*) of 5 – 7 Mya was used to represent the potential split time between both lice (Light et al., 2010; Ashfaq et al., 2015); (iii) *P*. *schaeffi* was estimated that the divergence time was 12 Mya for *cox*1 gene (Ashfaq et al., 2015). The *L*. *bostrychophila* was then set as the outgroup of whole taxa to calibrate the height of the BI tree and the mean age was set as 52 Mya. The minimum age was set up at 5 Mya using an exponential distribution. Each analysis was run for 40 million generations with sampling every 1000 generations. Tracer v1.7.2 was applied to monitor the convergence between runs the effective sample size (ESS) should be more than “200” (Rambaut et al., 2018). TreeAnnotator v2.6.2 was used to access the posterior probabilities of the maximum clade credibility (MCC) tree with the first 10% tree burn-in (Dellicour et al., 2021). The phylogenetic relationship and divergence time were visualized in FigTree v1.4.3.

## 3 Results

### 3.1 Taxonomy

Superfamily Trichodectoidea Kéler, 1938.

Family Trichodectidae Kellogg, 1896.

Genus *Pancola* **new genus**.

**Type species:***Pancola ailurus***new species**.

**Host distribution:** Carnivora: Ailuridae.

**Geographical range:** Himalaya-Hengduan Mountains.

**Diagnosis:***Pancola* is a new genus in the family of Trichodectidae. A dichotomous key by Lyal (1985) is used for the identification of *Pancola* with other genera in Trichodectidae. Based on the presence of exactly five abdominal spiracle openings, the abdominal chaetotaxy of both sexes, and the fact that the parameres are not fused to the lateral struts of the basal apodeme, *Pancola* is the closest to *Paratrichodectes*. However, these two genera can be separated by the following combination of characteristics: 1) the anterior head margin of the *Pancola* is broad, medium slightly convex, or non-convex, whereas the anterior of the head with osculum present in *Paratrichodectes* (Lyal, 1985); 2) the head marginal temporal carina with rounded edges in *Pancola*, differentiate it from *Paratrichodectes*, which has a convex or rectangular temple margin (Lyal, 1985); 3) the 6 pairs meso-metasternal setae present on *Pancola*, which is different from *Paratrichodectes*. There are no meso-metasternal setae on *Paratrichodectes* (Lyal, 1985); 4) the male genitalia of *Pancola* has narrow mesomeres, which is absent in *Paratrichodectes* (Lyal, 1985).

*Pancola* can be morphologically distinguished from other Trichodectidae by the following combination of characters: 1) anterior head margin broad, medium slightly convex or non-convex; frons sclerotized; marginal carina (*mc*) along the head margin and partially pigmented; 2) sexual dimorphic antennae present, the male antennae stouter and larger than which on female; 3) thorax with long meso-metasternal setae present; meso-metasternal plate small, X-shaped; 4) abdomen with long setae present on tergites and sternites; five pairs of abdominal spiracles present on segments III-VII, small in size; 5) male subgenital plate present, sclerotised; mesomeres present, apices of mesomeres fused; parameres tapering, separate to each other; symmetric; small spinose sac present; basal apodeme elongated, straight but slightly diverging; and 6) ventral terminalia with small tapered gonapophyses VIII in female, the inner margin of gonapophyses VIII gently convex, the outer margin of gonapophysis VIII within abdomen boundary.

**Fig. 2:**
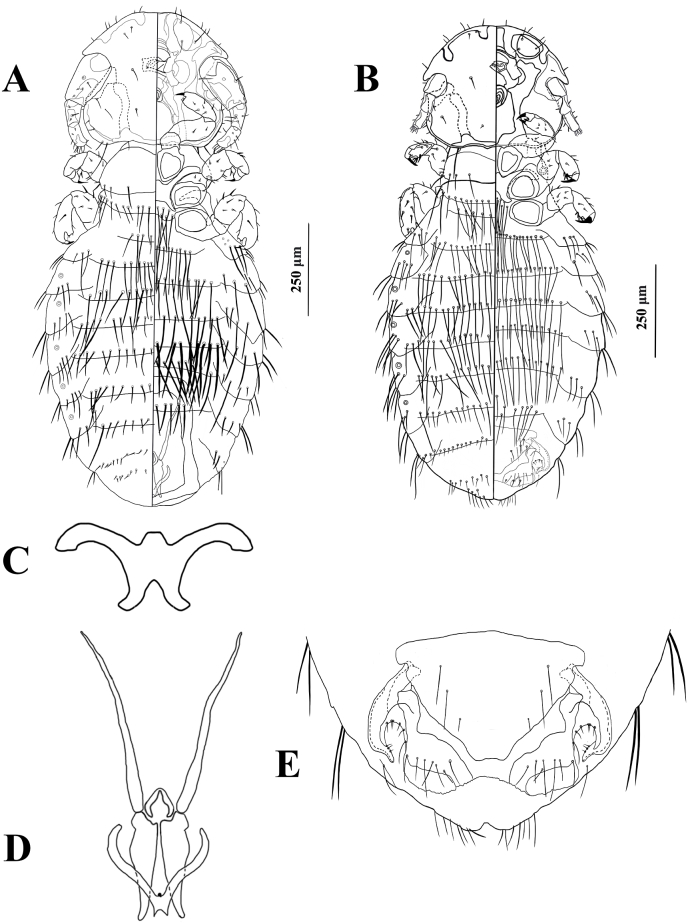
*Pancola ailurus* n. sp. **(A)** Male *Pancola ailurus* n. sp., habitus (dorsal morphology to the left of the midline, ventral morphology to the right) (**B**) Female *Pancola ailurus* n. sp., habitus (dorsal morphology to the left of the midline, ventral morphology to the right) (**C**) Meso-metasternal plate of *Pancola ailurus* n. sp. (**D**) Male genitalia **(E)** Female genitalia.

**Description. *Both sexes*.** Head rounded in shape. Pre-antennal region was broad, with slightly convex or non-convex sclerotized frons. Marginal carina (*mc*) partially pigmented; pre-marginal carina (*prmc*) continuous with post-marginal carina (*pomc*); preantennal nodus (*pran*) large, blunt. Antennae sexual dimorphic. Male antennae scape stout and larger than those in females. Antennae with 3 segments, antennal groove slightly deep; pedicel and flagellomere unfused, pedicel smaller than scape; flagellomere with the large process, slightly curved. Gular plate absent; temple margin convex; marginal temporal carina with rounded edges. Head chaetotaxy as in Fig. 2A and B. Dorsally, anterior seta 1 (*as1*), anterior seta 2 (*as2*), anterior seta 3 (*as3*), preconal seta (*pcs*), anterior dorsal seta 1 (*ads1*), anterior dorsal seta 2 (*ads2*), and preantennal seta (*pas*) present on each side; Ventrally, anterior ventral seta 1 (*avs1*), anterior ventral seta 2 (*avs2*), anterior ventral seta 3 (*avs3*), and mandibular (*mds*) present on each side. One pair of postnodal seta (*pns*), ocular setae (*os*), post-ocular setae (*pos*), and post-temporal setae (*pts*) on each side. Five pairs of marginal temporal setae (*mts*) on each side, vary in size, mts 4 large. Thoracic segments as in Fig. 2A and B. Prothorax undivided. Posterolateral setae present. Pterothorax short, posterior margin slightly convex. Pteronotum undivided, pterothoracic thorn-like setae (*pths*), pterothoracic trichoid seta (*ptrs*), and posterolater setae present. Meso-metasternal plate is small and X-shaped. Meso-metasternal setae present, long in size. Mesothoracic and metathoracic legs sternocoxal in articulation. Abdominal segments as in Fig. 2A and B. Wider than thorax, oval to rounded in shape. Dorsally, 5 pairs of spiracles in small sizes are present on segments III-VII. Abdominal chaetotaxy as in Fig. 2A and B. Tergal posterior setae (*tps*), and sternite setae (*sts*) are present, all setae long in size. Tergopleurite plates with paratergal seta (*ps*) and post-spiracular seta (*pss*) present.

**Male.** As in Fig. 2D. The subgenital plate present, is sclerotised; narrow mesomeres present, apices of mesomeres fused; parameres tapering, separate to each other; symmetric; small spinose sac present; basal apodeme elongated, straight but slightly diverging, longer than parameres.

**Female.** As in Fig. 2E. Ventral terminalia with small tapered gonapophyses VIII in females, the inner margin of gonapophyses VIII gently convex with marginal setae present, the outer margin of gonapophysis VIII within the abdomen boundary.

**Etymology:** The genus name is a noun referring to the common name of the host.

*Pancola ailurus***new species**.

**Type host:***Ailurus fulgens styani* Thomas, 1902 – red panda (Carnivora: Ailuridae).

**Type locality:** Species were collected in the Chengdu Research Base of Giant Panda Breeding, Chengdu County, Sichuan Province, China (N30.743°, E104.150°, 528 m above sea level (a.s.l)).

**Male** (n = 2). As in Fig. 2A, C, D. Body length 1.39 – 1.57 mm (mean 1.46 mm).

**Head:** As in Fig. 2A. Roughly round in shape, slightly wider than long. Pre-antennal Region: Anterior head margin broad, medium slightly convex or non-convex; frons sclerotized; *mc* along the head margin and partially pigmented; *prmc* continuous with *pomc*; *pran* large, blunt; conus blunt, smaller than the posterior end of scape in male and female. Dorsally, 1 pair of *as1*, *as2*, *as3*, *pcs*, *ads1*, *ads2*, and *pas* present on each side.; Ventrally, 1 pair of *avs1*, *avs2*, *avs3*, and *mds* on each side. Antennal Region: Antennae sexual dimorphic. Male antennae scape stout and larger than females. Antennae with 3 segments, antennal groove slightly deep; pedicel and flagellomere unfused, pedicel smaller than scape; flagellomere with the large process, slightly curved. Post-antennal Region: Gular plate absent; the margin of temple convex; marginal temporal carina with rounded edges. One pair of *pns*, *os*, *pos*, and *pts* on each side. Five pairs of *mts* on each side, vary in size, *mts* 4 large.

**Thorax:** As in Fig. 2A. Prothorax undivided. Posterior margin of pronotum with 4 pairs of posterolateral setae. Mesothoracic spiracles ventrolateral, small in size (0.24 – 0.32 mm). Pterothorax short, posterior margin slightly convex. Pteronotum undivided, dorsolateral margin with 1 pair of *pths*, *ptrs*, and 11 pairs of posterolater setae on each side. Meso-metasternal plate is small, X-shaped (Fig. 2C). Meso-metasternal setae 6 pairs, very long. Mesothoracic and metathoracic legs sternocoxal in articulation.

**Abdomen:** As in Fig. 2A. Wider than the thorax, oval to rounded in shape. Dorsally, 5 pairs of spiracles in small sizes are present on segments III-VII. One tergite per segment, except segments I and VIII without tergites and segment II with 2 tergites. Tergite 1 with 4 pairs of long *tps*, medium pair smaller. Tergites 2 and 3 each with 9 pairs of long *tps*. Tergites 4 and 5 each with 10 pairs of *tps*, medium pair very small. Tergite 6 with 9 pairs of long *tps*, medium pair very small. Tergite 7 with 9 pairs of *tps*, lateral pair large but medium pair very small. Segment 8 with 2 rows of setae, 1st row with 11 pairs of very small setae, 2nd row with 8 pairs of very small setae. Ventrally, six sternites with *sts* present from segment II to VII, all setae long in size. Sternite 1 with 17 long *sts*. Sternite 2 with 9 pairs of long *sts*. Sternite 3 and 4 with 8 pairs of long *sts*. Sternite 5 with 15 long *sts*. Sternite 6 with 5 pairs of long *sts*. Tergopleurite II with 2 pairs of *ps* and 3 pairs of *pss* on each side. Tergopleurites III to VI with a small spiracle, 2 pairs of *ps*, and 4 pairs of *pss* on each side. Tergopleurite VII with a very small spiracle, 2 pairs of *ps*, and 3 pairs of *pss* on each side. Tergopleurite VIII has no spiracles, with 2 small pairs of *ps* and 2 pairs of *pss* on each side.

**Male Genitalia:** As in Fig. 2D. Elongated, exceeds up to segment VI; male subgenital plate present, sclerotised; narrow, heart-like mesomeres present, apices of mesomeres fused with concave margin; parameres tapering, thicker and longer than mesomeres; small accessory structures at the base of parameres; small O-shaped spinose sac; basal apodeme elongated, straight but slightly diverging, V-shaped, much longer than parameres.

**Female:** (n = 1). As in Fig. 2B, E. Body length 0.97–1.56 mm (mean 1.33 mm).

**Head:** As in Fig. 2B. Much as in males, except for the antennae parts, female antennae scape much slimmer and shorter than in males. Antennae scape in female stout and elongated with the lateral process; pedicel and flagellomere unfused, pedicel smaller than scape; flagellomere with the process, no curve.

**Thorax:** As in Fig. 2B. As much as in males.

**Abdomen:** As in Fig. 2B. Wider than the thorax, oval to rounded in shape. Dorsally, 5 pairs of spiracles in small sizes are present on segments III-VII. One tergite per segment, except segments I and VIII without tergites and segment II with 2 tergites. Tergite 1 with 9 long *tps*, equal in size. Tergites 2 with 25 long *tps*. Tergite 3 with 13 pairs of long *tps*. Tergite 4 with 15 pairs of long *tps*. Tergite 5 with 14 pairs of long *tps*. Tergite 6 with 13 pairs of long *tps*. Tergite 7 with 12 pairs of *tps*, varies in size. Ventrally, six sternites with *sts* present from segment II to VII, all setae long in size. Sternites 1 and 2 each with 27 sts. Sternites 3 – 5 each with 12 pairs of *sts*. Sternite 6 with 8 pairs of *sts*. Tergopleurite II with 2 pairs of *ps* and 4 pairs of *pss* on each side. Tergopleurite III with a small spiracle, 2 pairs of *ps*, and 4 pairs of *pss* on each side. Tergopleurites IV and V each with a small spiracle, 2 pairs of *ps*, and 3 pairs of *pss* on each side. Tergopleurite VI with a very small spiracle, 2 pairs of *ps*, and 4 pairs of *pss* on each side. Tergopleurite VII with a very small spiracle, 2 pairs of *ps*, and 3 pairs of *pss* on each side. Tergopleurite VIII with no spiracles, 2 small pairs of *ps*, and 2 pairs of *pss* on each side.

**Female genitalia:** As in Fig. 2E. The subgenital plate is broad and subtriangular-shaped with three pairs of setae, apical pair setae shortest, other two pairs equal in size; the terminal portion of the subgenital plate without apical setae; two projections bordering the concavity at the apex with 4 pairs of setae on each projection; ventral terminalia with small tapered gonapophyses VIII, the inner margin of gonapophyses VIII gently convex with 9 marginal setae each side; the outer margin of gonapophysis VIII within abdomen boundary.

**Type material:** Holotype ♂ ex *Ailurus styani*, Chengdu Research Base of Giant Panda Breeding, Chengdu County, Sichuan Province, China (N30.743°, E104.150°), November 2020 (accession no. IOZ (E) 22144). Paratypes: 1♂, 1♀, same data as for the holotype and deposited in National Animal Collection Resource Center, China (accession no. IOZ (E) 221442, IOZ (E) 221443).

**Etymology:** The species epithet is a noun in apposition referring to the species scientific name of the host.

### 3.2 DNA sequence data

The amplified mt *cox*1 sequences showed 69.2 – 72.0% identities with species from genera *Geomydoecus* (KX228450), *Bovicola* (MH001203), *Damalinia* (JN122004), and *Trichodectes* (MH001214) species, and 12S rRNA gene sequences ranged from 75.3 – 85.3% with genera *Geomydoecus* (MW396892), *Bovicola* (MH001211 and MH001186), *Damalinia* (JN122002), and *Trichodectes* (MH001222), and 18S rRNA gene sequences of *P. ailurus* by conventional PCR showed 98.8 – 99.6% similarity with *Bovicola* (AY077769), *Felicola* (AY077770), and *Geomydoecus* (HQ124283) (Table S1).

### 3.3 Phylogenetic analyses and divergence time

**Fig. 3:**
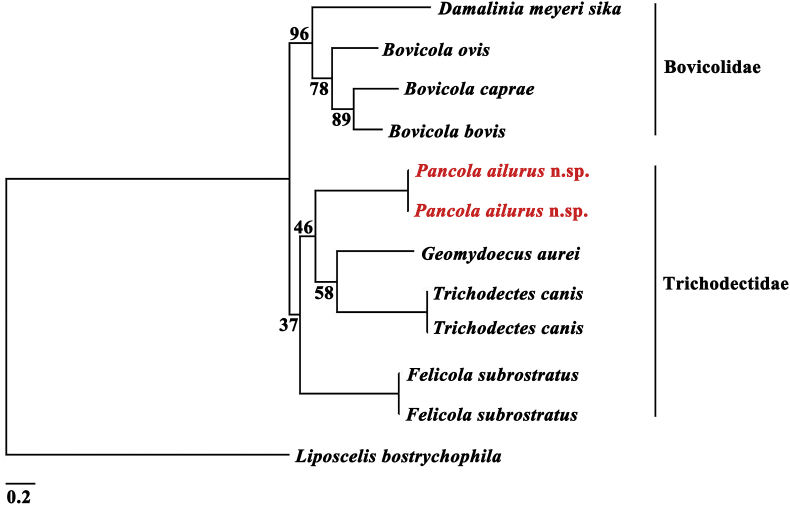
Phylogenetic trees based on the partial mitochondrial (*cox*1 and 12S rRNA) sequences of Trichodectidae and Bovicoliidae species using Maximum Likelihood (ML). The bootstrap frequencies (Bf) were shown on each node.

**Fig. 4:**
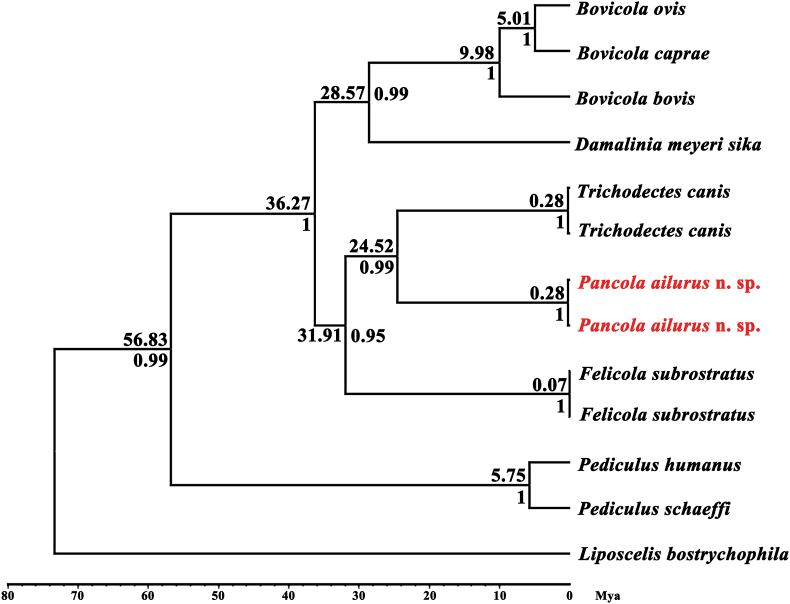
Divergence time and Bayesian analysis based on the partial *cox*1 sequence of Trichodectidae and Bovicoliidae species using Beast v.1.10.4 with *Liposcelis bostrichophila* as the outgroup.

We combined the mt *cox*1 and 12S rRNA genes for further phylogenetic analyses. All *cox*1 and 12S rRNA genes of specimens were individually aligned by ClustalX 1.83 (Aiyar, 2000), respectively. The mt genes phylogenetic reconstructions revealed similar topologies (Fig. 3, Fig. 4), and strongly supported the monophyly of the family Trichodectidae lineage. Among phylogenetic analyses, the family Trichodectidae clade was comprised of four genera *Trichodectes*, *Geomydoecus*, *Felicola*, and *Pancola*; the other obvious clade was the family Bovicoliidae, which mainly consisted of two genera *Bovicola* and *Damalinia*. The *P. ailurus* grouped the consistent topology with species *Felicola subrostratus*, *Geomydoecus aurei*, and *Trichodectes canis* with moderate support (Fig. 3), and had a closer relationship with *Trichodectes* and *Geomydoecus*.

Considering genetic divergence at the species level in various mammal groups was from 2 – 11% (Baker and Bradley, 2006), the distance for uncorrected *cox*1 and 12S rRNA genes among different species from families Trichodectidae and Bovicoliidae was 29.7 – 34.6% and 38.9 – 43.6%, respectively (Table 3), indirectly representing *P. ailurus* was the distinct species from genera *Felicola*, *Geomydoecus*, and *Trichodectes*.

**Table 3:** Uncorrected pairwise genetic distance of the mitochondrial genes (*cox*1 and 12S rRNA) sequences of chewing lice from families Trichodectidae and Bovicoliidae used in this study. The lower left represents the genetic distance of *cox*1, and the upper right represents the genetic distance of 12S rRNA.

Species	1	2	3	4	5	6	7	8
*Geomydoecus aurei*		0.374	0.438	0.466	0.445	0.464	0.458	0.428
*Trichodectes canis*	0.288		0.432	0.453	0.432	0.445	0.447	0.389
*Felicola subrostratus*	0.310	0.306		0.361	0.335	0.421	0.359	0.425
*Bovicola caprae*	0.305	0.310	0.330		0.192	0.253	0.361	0.413
*Bovicola bovis*	0.312	0.321	0.314	0.218		0.268	0.335	0.426
*Bovicola ovis*	0.305	0.302	0.314	0.272	0.236		0.382	0.426
*Damalinia meyeri sika*	0.333	0.333	0.346	0.312	0.318	0.322		0.436
*Pancola ailurus*	0.300	0.297	0.322	0.322	0.312	0.310	0.346	

Divergence analysis of *cox*1 alignment supported a clear separation with *Pancola*, *Felicola*, and *Trichodectes* within the family Trichodectidae, and dated the split time within them at ∼24.52 Mya (with 95% highest posterior density, HPD, 15.93–35.52 Mya) (Fig. 4). The topology of divergence analysis was the same as ML analysis. In all analyses, disregarding other unknown molecular data, the *cox*1 gene sequences from families Trichodectidae and Bovicoliidae were grouped into two branches separated from primate lice, *Pediculus humanus* and *P. schaeffi*. The divergence tree further indicated that associated divergence time within the family Trichodectidae (*Felicola*, *Trichodectes*, and *Pancola*) from 24.52 Mya (95% HPD, 15.93–35.52 Mya) to 31.91 Mya (95% HPD, 21.40–45.98 Mya), and the origin of *Pancola* was closer to *Trichodectes*.

## 4 Discussion

We describe one new species, *Pancola ailurus* n. sp., from Chinese red pandas. This description increases the number of known genera of Trichodectoidea from 19 to 20. First, the morphological features that distinguished those species have been identified as above-described. Second, results showed a phylogenetic relationship within the family Trichodectiidae with a divergence time of 24.52 – 31.91 Mya (Fig. 4) and genetic distances greater than 29.0% (Table 3), which is higher than species-level (Baker and Bradley, 2006), supporting that *Pancola* was a distinct genus, and *P. ailurus* was a validate species. Consistent with hosts divergence and phylogenetic analyses that canids are closer to red pandas (Agnarsson et al., 2010; Nyakatura and Bininda-Emonds, 2012), relationships and the origin between *Trichodectes* and *Pancola* species were more related. However, Lyal (1983) proposed *Trichodectes* was more related to *Felicola*, but a relationship based on mt genes showed *Trichodectes* may be closer to *Geomydcecus*.

As *L*. *bostrychophila* was added as the outgroup with *cox*1 for 100 Mya and the estimated split time for human lice (*P. humanus*) and chimpanzee lice (*P. schaeffi*) was 5–7 Mya for *cox*1, the divergence time was estimated for *Bovicola*, *Damalinia*, *Trichodectes*, *Felicola*, and *Pancola* approximately ranging from 5.01 Mya to 36.27 Mya for *cox*1 gene. Similar to Light et al. (2010) analyses that the divergence time of the same genus was equal to/less than 15 Mya, the divergence time among *Bovicola* species (*B. ovis*, *B. caprae*, and *B. bovis*) was from 5.01 to 9.98 Mya, and *Pediculus* species diverged to *P. schaeffi* and *P. humanus* about 5.75 Mya.

Based on the morphological keys of Lyal (1985), the genus *Pancola* is probably closely related to *Paratrichodectes*. However, available sequences are absent from a large number of trichodectid genera, including *Paratrichodectes*, and no direct comparisons could be made. The correct placement of *Pancola* within Trichodectidae will therefore have to be evaluated when more sequences of different trichodectid genera are available. Additionally, overall phylogenetic relationships of Trichodectidae using molecular information were similar to Lyal (1983) and Kéler (1938) observations, suggesting that molecular markers could be a useful supplemented method to identify lice.

### 4.1 Nomenclatural acts registration

**ZooBank registration:** To comply with the regulations set out in the article 8.5 of the amended 2012 version of the International Code of Zoological Nomenclature (ICZN), details of the new species have been submitted to ZooBank.

The Life Science Identifier (LSID) of the article is urn:lsid:zoobank.org:pub:C188B59C-6258-4681-90DB-60CA8B0B3690.

The LSID for the new genus name *Pancola* is urn:lsid:zoobank.org:act:A4CB7AFD-AE04-4E48-9B61-2D20E5E30BD9.

The LSID for the new species name *Pancola ailurus* is urn:lsid:zoobank.org:act:22FDB0D6-1121-45C1-8CD7-B1D3860B42E7.

## Authors’ contributions

G.H.L. and Y.P.D. designed and supervised the study; Y.P.D. carried out all analyses and wrote the manuscript; W.W. conducted the morphological analyses and drew the morphological figures. Y.P.D. and G.H.L. analyzed the molecular data and accomplished the preliminary manuscript. Y.T.F. and Y.N. submitted the sequences to GenBank. Y.X. collected the samples. Y.P.D., W.W., Y.X. and G.H.L. revised the manuscript. All authors read and approved the final version of the manuscript.

## Data availability statement

The sequence data is uploaded to the NCBI GenBank and the raw sequences are available under the accession of ON964542, ON973802, ON964865, ON964866, and ON964867.

## Author statement

The authors declare that they have no known competing financial interests or personal relationships that could have appeared to influence the work reported in this manuscript.

## Declaration of competing interest

The authors declare that they have no competing interests.
