# Supplementary material for: Morphological and molecular evidence reveals a new species of chewing louse Pancola ailurus n. sp. (Phthiraptera: Trichodectidae) from the endangered Chinese red panda Ailurus styani
Source: Int J Parasitol Parasites Wildl. 2022 Dec 27;20:31–8. doi: 10.1016/j.ijppaw.2022.12.004 (PMC9811220; doi:10.1016/j.ijppaw.2022.12.004)
Supplement: Multimedia component 1 [file mmc1.docx]

Table S1 The identities among *Pancola ailurus* with other Trichodetidae species.

| Species  Gene | *Geomydoecus* | *Bovicola* | *Damalinia* | *Felicola* | *Trichodectes* |
| --- | --- | --- | --- | --- | --- |
| *cox*1 | 71.8% | 72.0% | 69.2% | - | 71.5% |
| 12S rRNA | 76.6% | 85.3% | 81.8% | - | 77.6% |
| 18S rRNA | 98.8% | 99.6% | - | 98.8% | - |
